# Ossified chronic epidural hematoma of the posterior fossa

**DOI:** 10.11604/pamj.2015.20.238.3954

**Published:** 2015-03-13

**Authors:** Ali Akhaddar, Omar Boulahroud

**Affiliations:** 1 Department of Neurosurgery, Avicenne Military Hospital, Marrakech, Morocco; 2 University of Mohammed V Souissi, Rabat, Morocco; 3 Department of Neurosurgery, Mohammed V Military Teaching Hospital, Rabat, Morocco

**Keywords:** Chronic, epidural hematoma, posterior fossa

## Image in medicine

A 15-year-old adolescent presented to us with mild headache, progressive dizziness and recent vomiting since 2 weeks without seizures. There was a history of head injury (neglected) following a stone-throwing incident 2 months earlier. On examination, he was conscious with a left cerebellar syndrome but without other neurologic deficit. Routine biologic data were normal. CT-scan revealed left posterior fossa extradural lesion with hypodensity in the center and calcified wall (A). MRI showed that the lesion was liquid with homogeneous ring enhancement following gadolinium injection and cerebellar compression (B and C). A sub-occipital craniotomy was done and a chronic liquefied hematoma was removed. There was a thick hard calcified wall relatively adherent to the duramater (D). Postoperative period was uneventful and the patient was discharged symptoms free. Histologically, the wall contains large areas of ossification. Ossified chronic epidural hematoma is a very rare complication following head injury. The blood accumulates slowly from a venous source becomes chronic extradural hematoma and becomes calcified or ossified due to an inflammatory reaction of the dura especially in children. This rare phenomenon should be considered in the differential diagnosis of other traumatic epidural and subdural hematomas.

**Figure 1:**
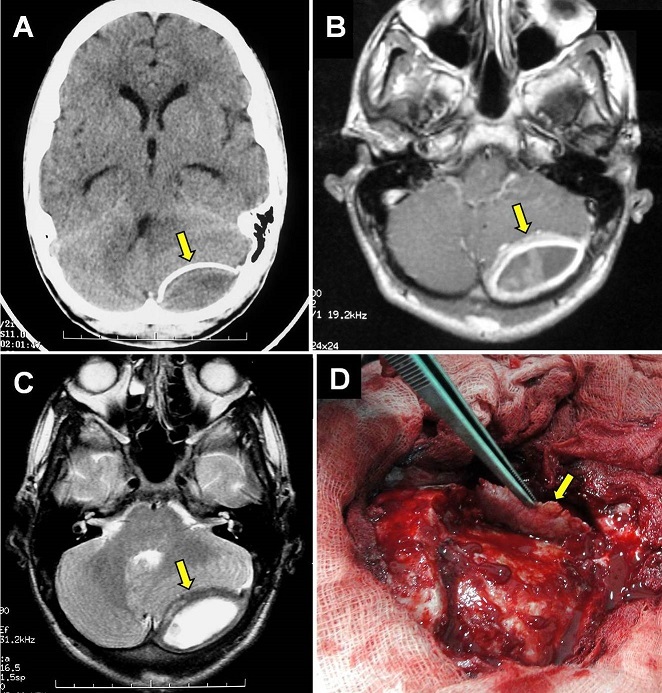
Axial CT-scan. (A): revealing a left posterior fossa chronic extradural biconvex lesion with ossified wall (arrow). MRI: Axial T1-weighted image after gadolinium injection; (B): T2-weighted image; (C): fluid-liquid lesion with homogeneous ring enhancement and significant cerebellar compression; (D): operative view demonstrating the ossified wall (arrow) after superficial dissection from the duramater

